# Microencapsulation, Physicochemical Characterization, and Antioxidant, Antibacterial, and Antiplasmodial Activities of *Holothuria atra* Microcapsule

**DOI:** 10.1155/2024/5559133

**Published:** 2024-06-14

**Authors:** Prawesty Diah Utami, Herin Setianingsih, Dewi Ratih Tirto Sari

**Affiliations:** ^1^Parasitology Department, Faculty of Medicine, Hang Tuah University, Surabaya, Indonesia; ^2^Anatomy and Histology Department, Faculty of Medicine, Hang Tuah University, Surabaya, Indonesia; ^3^Pharmacy Department, Faculty of Health Science, Ibrahimy University, Situbondo, Indonesia

## Abstract

This study provides the design of a microencapsulation formula, physicochemical characterization, and antioxidant, antibacterial, and antiplasmodial activities of *Holothuria atra* microcapsules. The ethanolic extract of *H. atra* was microencapsulated with chitosan (CHI) and sodium tripolyphosphate (Na-TPP) with various stirring times: 60 minutes (CHI60), 90 minutes (CHI90), and 120 minutes (CHI120). The microcapsules were then observed for physicochemical properties using scanning electron microscopy (SEM) and Fourier-transform infrared spectroscopy (FTIR). The microcapsules were tested for antioxidant activity and antibacterial activity against *Staphylococcus aureus* and *Escherichia coli* using the DPPH (2,2-diphenyl-1-picrylhydrazyl) method. Antiplasmodial bioactivity was assessed through in silico molecular docking. The CHI60 and CHI120 microcapsules exhibited a smaller size and an irregular spherical shape, while the same FTIR profile was observed in CHI90 and CHI120. The bioactivity tests demonstrated that CHI90 exhibited high antibacterial activity against *E. coli* and *S. aureus*, while CHI120 exhibited high antioxidant performance. Calcigeroside B and Echinoside B exhibited antiplasmodial activity against the *Plasmodium falciparum* dihydroorotate dehydrogenase (PfDHODH) protein, along with an artemisinin inhibition mechanism. In conclusion, the microcapsules with the CHI90 formula demonstrated the best antibacterial activity, while the CHI120 formula exhibited high antioxidant activity. Two terpenoids, Calcigeroside B and Echinoside B, exhibited the best antiplasmodial activity.

## 1. Introduction

The utilization of organic bioactive substances has experienced significant growth in several industries, including food, fabric, skincare products, fragrances, and pharmaceutical products, owing to their exceptional bioactive properties. The sea cucumber is widely recognized as a very promising marine organism due to its abundant organic bioactive substances and diverse range of biological activity [1]. This organism is classified within the phylum Holothuroidea, which is a part of the larger taxonomic group known as Echinodermata. The aquatic environment harbors a species of organisms that bear a resemblance to cucumbers. Out of an estimated global population of 1,200 species, 66 species under the taxonomic category of Holothuroidea have been classified as sea cucumbers [2]. The potential health benefits of *Holothuria atra* biological substances include protection from cardiovascular disease, antidiabetic properties, hypoglycemic effects, antioxidant activity, antiasthmatic properties, antieczema effects, anti-inflammatory effects, cholesterol-lowering effects, immunomodulatory effects, cytotoxic properties, antiparasitic activity, antimalarial activity, antiviral effects, antifungal properties, antiangiogenic effects, and anticancer effects [3, 4].

However, the inherent instability and suboptimal bioavailability of these substances restrict their ability to exert their biological functions [5]. Hence, the utilization of appropriate carriers for encapsulation becomes highly significant. Currently, there is a trend toward encapsulating bioactives to create fibrous and capsular structures that have a wide range of applications. The utilization of encapsulation techniques has been observed to effectively address the limitations associated with the delivery of bioactive substances to specific target areas [6].

Microencapsulation is a viable technique utilized for the purpose of delivering various types of components in their functional states to specific regions inside a biological system. The encapsulation procedure refers to a technological method wherein a substance or combination of substances is enclosed within micro and/or nanostructures by entrapping a bioactive core with another substance known as wall materials [7, 8]. This process serves to safeguard bioactive substances, such as flavors and pigments, among others. The composition of the microcapsules includes the core and shell. The core is the substance that is encapsulated, while the shell is the polymer utilized for encapsulating bioactive substances. The wall material exhibits no chemical reactivity with the enclosed substance [9]. Effective strategies can be employed to encapsulate active substances, safeguarding them against adverse conditions such as high temperatures, oxidation, alkaline or acidic environments, moisture, or evaporation. The conversion of liquids into powder form is a viable method for enhancing the process of encapsulating biological substances, as it helps prevent the formation of clumps in the final product. The transition from a liquid to a solid state also serves to limit the interaction of these substances with undesired species that could potentially induce polymerization inside the reaction mixture [10]. The selection of an encapsulating material is contingent upon the maintenance of structural integrity, which is a crucial factor in determining the efficacy of shell materials. The processing and economic dimensions are also included in this matter. The selection of microcapsules for encapsulation is influenced by several pertinent factors, including toxicity degree, efficacy, stability, protective level, and microscopic features [5, 9].

Chitosan is a biopolymer with cationic properties that is found in abundance following cellulose. It is characterized by the presence of several amino and hydroxyl groups. Chitosan exhibits notable hydrophilic properties due to the presence of amino groups that carry a positive charge. The polycationic properties of chitosan have garnered significant commercial importance due to its potential use in a wide range of applications [11]. The application of this coating on shells for encapsulating materials has proven to be quite beneficial. Chitosan-coated encapsulated products containing active substances have been demonstrated to possess enhanced functional characteristics [8]. Chitosan finds widespread utilization in microencapsulation across various domains, including biomedical research, the agricultural sector, skin care products, food, and fabrics.

Novel formulations, particularly microencapsulation of *H. atra*, need to be examined for pharmaceutical purposes, involving the exploration of new drugs, assessment of their binding properties and specificity, characterization of their molecular structures, and confirmation of their efficacy and safety. Physicochemical characterization is important for predicting constituent behavior, assessing performance, and ensuring formulation stability in the development of new compounds and formulations. Methods such as electron microscopy and spectrophotometry are employed to regulate the function of an ingredient in the formulation and confirm the accurate distribution of ingredients [12]. Assessing antioxidant activity in novel materials is crucial for assessing their ability to reduce oxidative stress and protect materials from degradation. The DPPH (2,2-diphenyl-1-picrylhydrazyl) approach is a commonly utilized methodology for assessing the antioxidant properties of different materials, including novel ones [13]. Antibacterial analysis is conducted on novel substances to assess their capacity to impede the growth of bacteria and avert the transmission of infections. The evaluation is crucial to ascertain the efficacy of novel substances in avoiding the emergence of resistance to antibiotics and minimizing bacterial infections [14, 15]. The disc diffusion technique is a commonly employed antibacterial test assay that assesses the sensitivity of bacteria to antimicrobial drugs [16]. An antiplasmodial analysis aims to assess the capacity of a substance to hinder the growth of *Plasmodium*, a parasite causative of malaria. The test is designed to assess the antiplasmodial action of chemicals, extracts, or substances, playing a crucial role in identifying and creating new antimalarial remedies. Antiplasmodial action can be assessed by in silico, in vitro, or in vivo techniques [17, 18]. In silico approaches utilize computer simulations and modeling to forecast the prospective antiplasmodial action of substances [18].

Based on these phenomena, this study involved conducting an encapsulation procedure on the *H. atra* extract using chitosan with different periods of condensation. This study provided microencapsulation and antioxidant, antibacterial, and antiplasmodial activities of the *H. atra* ethanolic extract.

## 2. Methods

### 2.1. Sample Collection and Preparation

Fresh *H. atra* specimens were collected from the waters of Sapeken Island, Sumenep, Madura, Indonesia, in April 2023 (dry season). The internal organs were carefully removed, cleaned, and then sun-dried (32–34°C) for 3 days. The dried *H. atra*, with approximately <10% water content, was collected and ground using a grinder machine. The powder was filtered using a sieve with 40 meshes to obtain a fine powder of *H. atra.* Five kilograms of dried whole *H. atra* yielded 4,358 g of *H. atra* fine powder.

### 2.2. Extraction and Formulation of *Holothuria atra* Microcapsules

Freeze-drying, a prevalent method for extracting water from a substance while maintaining its structure and composition, is a component of the process outlined. This method prevents the sample from being damaged by high temperatures, which is especially advantageous when dealing with heat-sensitive substances such as biological samples or fragile microcapsules [19]. Approximately 1 kg of *H. atra* fine powder was soaked with 2 L of ethanol (Merck, Germany) overnight. The solution was filtered using filter paper and evaporated using a rotary evaporator at 70°C and 500 rpm. The residues were macerated and evaporated, and the dried extract was prepared for encapsulation. The encapsulation of *H. atra* was processed by ionic gelation processes with chitosan–tripolyphosphate [20]. The *H. atra* extract (0.5 g) was dissolved in 17.5 mL of deionized water. The extract solution was mixed with 50 mL of 1% chitosan in 2% acetic acid buffer. The mixture was stirred for various times, specifically 60, 90, and 120 minutes at 500 rpm. Sodium tripolyphosphate (Na-TPP) at 0.3% (175 mL) was slowly added to the mixture. The microcapsule solution was subsequently dried using freeze-drying at −55°C and a pressure of 0.02 mbar for 96 hours. A fine powder of the microcapsules was obtained by grinding the sample using a mortar and pestle.

### 2.3. Physicochemical Characterization

Physicochemical characterization is of utmost significance in the pharmaceutical sector, where it is utilized to guarantee the efficacy, safety, and quality of pharmaceutical products [12]. Our study employed scanning electron microscopes (SEM) and Fourier-transform infrared spectra (FTIR) to assess physicochemical properties. FTIR spectroscopy is a method that captures the infrared spectrum of emission or absorption from a material, whether it is a liquid, gas, or solid [21]. SEM is a high-resolution imaging method that analyzes the surface features and chemical structure of materials using advanced equipment to observe the microstructure of objects [22].

The morphology of samples was observed using the TM3000 Olympus Tabletop SEM with 8,000–12,000x magnifications. Briefly, the microcapsule powder was dropped onto a chip coated with Au (gold) metal, air-dried, and observed on the scanning electron microscope. To identify the functional group of *Holothuria atra* microcapsule, FTIR was employed [23]. The *H. atra* microcapsule powder was placed on the ATR diamond crystal. Subsequently, both the sample and crystal were clamped using pressure gauges. The spectra were recorded using a Shimadzu FTIR spectrophotometer with attenuated total reflection (ATR-FTIR) in the range from 4000 cm^−1^ to 500 cm^−1^. The FTIR diagram was plotted using OriginPro 2021.

### 2.4. Antioxidant Activity of *Holothuria atra* Microcapsules

The DPPH method was selected to assess antioxidant activity due to its simplicity, quickness, and capacity to accurately measure antioxidant capacity. It is advisable to utilize many strategies to thoroughly comprehend antioxidant activity, as there are alternative approaches beyond this one [13]. The antioxidant activity of *H. atra* microcapsules was determined by the DPPH method with some modifications [24]. One milliliter of 0.1 g/mL microcapsules was mixed with a 50 mM DPPH solution in methanol. The mixture was incubated at 37°C in dark conditions for 30 minutes. The absorbance was observed using a spectrophotometer with a wavelength of 517 nm. Methanol was employed as a blank, and a DPPH solution without the sample was employed as a control.

### 2.5. Antibacterial Activity of *Holothuria atra* Microcapsules against *Staphylococcus aureus* and *Escherichia coli*

The antibacterial activity of *H. atra* microcapsules was tested using the disc diffusion method [16]. *S. aureus* and *E. coli* were selected for targeted bacterial inhibition activities. The bacterial cultures were obtained from the Faculty of Medicine, Brawijaya University, Malang, Indonesia. *S. aureus* and *E. coli* culture cells were inoculated in 0.9% sodium chloride, and then 200 *μ*L was spread on a nutrient agar medium. Sterilized and blank paper disks (6 mm diameter, MN Germany) were prepared and separately soaked in 250 mg/mL of amoxicillin and *H. atra* microcapsules with a concentration of 100 mg/mL. The paper disks were placed on the cell culture and incubated overnight at 37°C. The antibacterial activity of the microcapsules was determined by measuring the inhibition zone of the samples.

### 2.6. Antiplasmodial Activity of *Holothuria atra* Microcapsules

Nine terpenoid compounds were selected for modeling and antiplasmodial testing using an in silico approach. Assessing antiplasmodial activity in silico entails utilizing computational techniques to forecast a compound's ability to hinder the growth of *Plasmodium parasites* responsible for malaria [25]. The structures of nine terpenoids, namely, 17-hydroxyfuscocineroside B (CID 25099007), holothurin A3 (CID 71728339), holothurin A1 (CID 102057279), calcigeroside B (CID 163105984), fuscocineroside C (CID 44559164), holothurin B (CID 23674754), Echinoside A (CID 156831), 24-dehydroechinoside B (CID 101610324), and echinoside B (CID 73999936), were retrieved from the PubChem NCBI database. Chitosan, as a polymer for the microcapsule, was obtained from the structure available on PubChem NCBI. A plasmodial protein target, Plasmodium falciparum dihydroorotate dehydrogenase (PfDHODH) (PDB ID 6GJG), was downloaded from the protein database [26]. *H. atra* microcapsules were modeled using Hex 8.0 by interacting among terpenoids and bioactive compounds with chitosan. The terpenoids–chitosan complex was redocked into the PfDHODH protein using Hex 8.0 with parameters Shape + DARS [27]. The microcapsule–protein interactions were visualized and analyzed using Discovery Studio version 21.1.1.

## 3. Results and Discussion

**Figure 1:**
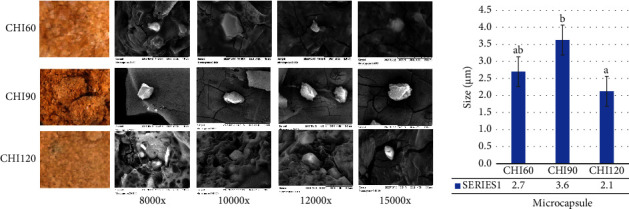
Morphological performances of *Holothuria atra* microcapsules with chitosan–Na-TPP with varied stirring time.

Malaria remains a significant global infectious disease with a high mortality and morbidity rate [28]. The search for new antimalarial agents has been a recent concern. These agents should be recognized for their safety and their ability not to develop resistance. In a previous study, *H. atra* exhibited antiplasmodial activity against *Plasmodium falciparum* [3]. However, the *H. atra* extract was not stable. An encapsulation technique was required to protect the compounds from environmental degradation. This study utilized a natural polysaccharide, chitosan, to microencapsulate the *H. atra* ethanolic extract. The mixture of the extract and polymer was stirred for different times, specifically 60, 90, and 120 minutes, to optimize the microencapsulation. The morphological character of *H. atra* microcapsules is presented in Figure 1. *H. atra* microcapsules have a brown color and exhibit a more intense brown with medium dryness at a stirring time of 90 minutes (CHI90). *H. atra* microcapsules with stirring times of 60 and 120 minutes exhibited similar textures and colors. Morphological characteristic observations with scanning electron microscopy revealed some microcapsules with an irregular shape. According to the SEM observation, *H. atra* CHI120 has the smallest microcapsules with a size of 2.1 *μ*m, followed by CHI60 and CHI90. Chitosan has been reported as a natural polymer for microencapsulating natural compounds [8, 11, 18, 19]. A previous report used chitosan to encapsulate cinnamon leaf oil and demonstrated irregular spherical shapes of microcapsules. The study also confirmed that the concentration of the extract and polymer affected encapsulation efficiency [29]. The microencapsulation steps in microcapsule formulation also affected the microcapsules' physical, chemical, and bioavailability. Adding sodium tripolyphosphate (Na-TPP) in stirring processes is critical to creating a stable emulsion [30]. The size of the microcapsules is also affected by NaCl and chitosan concentrations. Increasing NaCl and chitosan concentrations increased the particle size of the microcapsules. NaCl exhibited particle aggregation and competed against chitosan for association [31].

**Figure 2:**
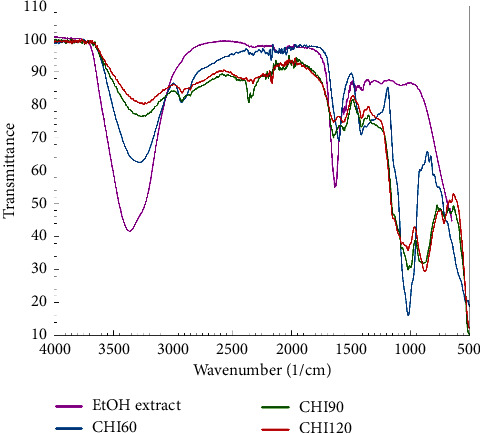
FTIR spectra of the *Holothuria atra* ethanolic extract and microencapsulation of the *H. atra* ethanolic extract with chitosan at stirring times of 60, 90, and 120 minutes.

The FTIR spectra of the *H. atra* ethanolic extract with various stirring times exhibited different profiles (Figure 2). The *H. atra* ethanolic extract and microcapsules demonstrated a broad hydroxyl group at 3,300 cm^−1^, and at 2,900 cm^−1^, the microcapsules exhibited a stretch peak. For a stirring time of 60 minutes (CHI60), closed peaks were observed, resembling those of the ethanolic extract of *H. atra.* Interestingly, CHI60 exhibited a stretch peak at 1,000 cm^−1^ that was not identified in all samples. CHI90 and CHI120 exhibited similar broad peaks at 1700 cm^−1^–1400 cm^−1^. Chitosan is a cationic polysaccharide of D-glucosamine and N-acetyl-D-glucosamine units linked by *β*-(1–4)-glycosidic bonds. Chitosan was reported to have a broad, intense hydroxyl group at 3,369 cm^−1^, 2,866 cm^−1^, 1,655 cm^−1^, and 1,550 cm^−1^ [19]. A previous study also reported that chitosan exhibited a strong band in the 3,291–361 cm^−1^ region, representing N-H and O-H stretching, in the 2,921 cm^−1^ region representing C-H symmetric stretching, and in the 2,877 cm^−1^ region representing C-H asymmetric stretching [32]. This study confirmed that stirring time in the microencapsulation process altered the wavenumber shift, indicating functional groups in the complex. CHI60 exhibited a sharp peak near 1,500 cm^−1^ that might be predicted as an electrostatic interaction of the ethanolic extract and chitosan.

**Figure 3:**
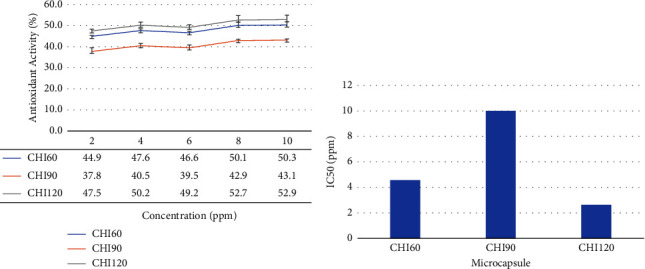
Antioxidant capacity (a) and inhibitory concentration (b) of *Holothuria atra* microcapsules.

Antioxidant activities were tested against DPPH as a free radical solution to assess the bioavailability of *H. atra* microcapsules. As depicted in Figure 3(a), CHI120 exhibited higher antioxidant activity than others. However, CHI60 also exhibited higher antioxidant activity than CHI90 and was close to CHI120. The antioxidant activity was negatively correlated with the inhibitory concentration (IC50) of *H. atra* ethanolic microcapsules. CHI120 exhibited the lowest IC50, followed by CHI60, and the last was CHI90 (Figure 3(b)).

**Figure 4:**
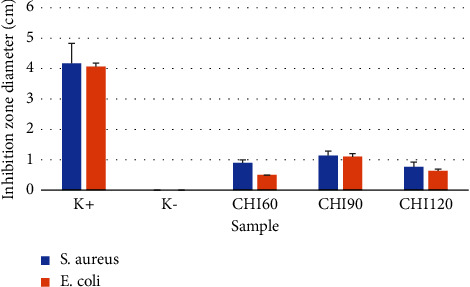
Antibacterial activity of the *Holothuria atra* ethanolic extract tested against *S. aureus* and *E. coli*.

The antibacterial performances of *H. atra* ethanolic microcapsules against *S. aureus* and *E. coli* are presented in Figure 4. CHI90 exhibited high antibacterial activity, inhibiting both *S. aureus* and *E. coli.* CHI60 and CHI120 demonstrated low activity against *E. coli* and high inhibition activity against *S. aureus* growth. In comparison, amoxicillin at 250 mg/mL demonstrated the highest antibacterial activity. The antibacterial activity of the *H. atra* extract has been reported in previous studies. The methanolic extract of *H. atra* inhibited *Vibrio alginolyticus* and *Vibrio anguillarum.* Moreover, 500 *μ*g/mL of the *H. atra* extract with ethyl acetate as a solvent demonstrated higher inhibition activity against *Vibrio alginolyticus* and *Vibrio anguillarum* than ampicillin at 10 *μ*g/mL [33]. In another study, the methanol and hexane fractions of *H. atra*, containing phenolic, terpenoids, and saponin compounds, exhibited antibacterial activity against *Pseudomonas aeruginosa* [34]. Chitosan, as a microcapsule polymer, has been reported to promote antibacterial activity against *Klebsiella pneumoniae*, *Pseudomonas aeruginosa, E. coli*, and *S. aureus* [35, 36]. Therefore, using chitosan as an encapsulation matrix might improve the antibacterial and antioxidant activity of *H. atra*.

The antiplasmodial performance of *H. atra* microcapsules was predicted through virtual prediction, involving the interaction of bioactive compounds with the dihydroorotate dehydrogenase of *P. falciparum* (*Pf*DHODH) [26]. Dihydroorotate dehydrogenase (DHODH) is being considered a therapeutic candidate due to its potency in the pyrimidine biosynthetic pathway. It plays a critical role in pyrimidine biosynthesis by facilitating Flavin mononucleotide-dependent formation of orotic acid [37–40]. As the pyrimidine biosynthetic pathway is crucial for cell growth, metabolism, and replication, DHODH has been identified as an important therapeutic target.

**Figure 5:**
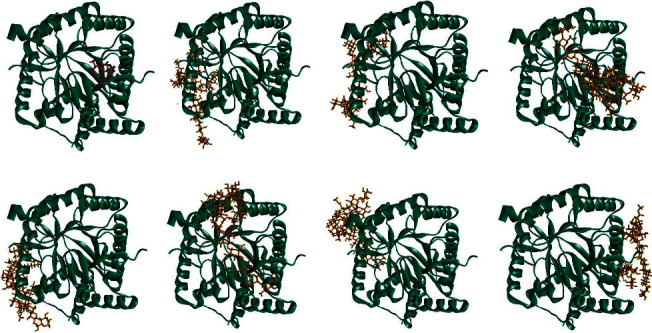
Binding poses of terpenoid microcapsules with PfDHODH: (a) artemisinin; (b) 17-hydroxyfuscocineroside B; (c) 24-dehydroechinoside B; (d) calcigeroside B; (e) echinoside A; (f) echinoside B; (g) fuscocineroside C; and (h) holothurin B. The PfDOODH protein is presented in a green cartoon, and terpenoid compounds of *Holothuria atra* are presented in yellow.

**Figure 6:**
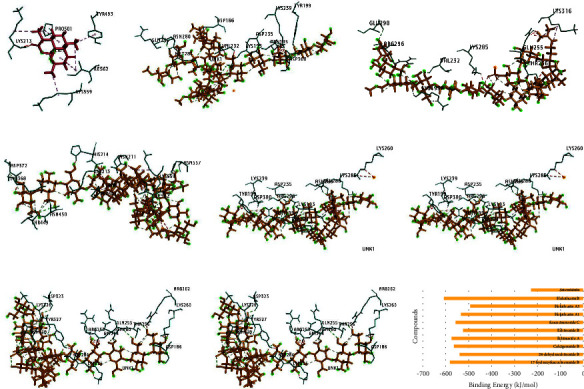
3D structure of the terpenoid microcapsule complex with the PfDHODH protein and binding energy of the complex: (a) artemisinin; (b) 17-hydroxyfuscocineroside B; (c) 24-dehydroechinoside B; (d) calcigeroside B; (e) echinoside A; (f) echinoside B; (g) fuscocineroside C; (h) holothurin B; and (i) binding energy of the ligands–protein complex.

Interactions of the bioactive compounds from *H. atra microcapsules* were compared with artemisinin as a control. Seven bioactive compounds of *H. atra* microcapsules, namely 17-hydroxyfuscocineroside B, 24-dehydroechinoside B, calcigeroside B, echinoside A, echinoside B, fuscocineroside C, and holothurin B, successfully formed bindings with *Pf*DHODH (Figure 5). All bioactive compounds from *H. atra* microcapsules demonstrated strong binding to DHODH, as indicated by lower binding energy compared to the *Pf*DHODH–Artemisinin complex (Figure 6). Considering the amino acid residues engaged in the binding process, Echinoside B and Calcigeroside B demonstrated binding to amino acid residues that closely resembled those involved in the binding of artemisinin. Calcigeroside B formed a complex with *Pf*DHODH involving amino acid residues Lys368, Lys559, Ile562, Asp372, Thr449, Asn557, Pro501, Asn211, and Lys213 through hydrogen bonds. Echinoside B is bound to DHODH through Asp235, Asn289, Asp300, Glu288, Lys193, Asn195, Tyr194, Tyr199, Lys260, Lys285, K239, and Arg296. Both Calcigeroside B and Echinoside B formed bindings to DHODH with similar amino acid residues, including Ile562, Pro501, Lys559, and Lys213. The similarities in amino acid residues indicating the potency of these compounds are comparable to those of artemisinin. According to binding energy, all seven terpenoid compounds exhibited lower binding energy than artemisinin, with Holothurin B having the lowest binding energy. A low binding energy indicates a tight interaction between ligands and proteins. The binding energy score is affected by the complexity of the ligand structure, the number of hydrogen bonds, and hydrophobic interactions [41–44].

## 4. Conclusion

The morphologies of microcapsules CHI60 and CHI120 exhibited a smaller size and an irregular spherical shape. CHI120 demonstrated high antioxidant activity, while CHI90 exhibited high antibacterial activity against *E. coli and S. aureus.* Calcigeroside B and Echinoside B exhibited antiplasmodial activity against the PfDHODH protein and bound to artemisinin sites. Furthermore, Calcigeroside B also demonstrated a lower binding energy, indicating a tight interaction between the compound and the PfDODH protein [45].

## Data Availability

The data used to support the findings of this study are available from the corresponding author upon request.
